# New magnetic nanocomposites based on hexafrite and keggin-type -type heteropolyanions: Synthesized and characterized for removal of environmental pollutants

**DOI:** 10.1016/j.heliyon.2024.e30289

**Published:** 2024-04-27

**Authors:** Mohammad Ali Rezvani, Amirhossein Hemmatzadeh, Mir Saeed Seyed Dorraji, Narges Nourbakhsh, Ghazal Oroumi

**Affiliations:** a Department of Chemistry, Faculty of Science, University of Zanjan, 451561319, Zanjan, Iran; b Department of Chemical Engineering, University of Tehran, Tehran, 1417935840, I.R. Iran, Iran

**Keywords:** Polyoxometalate, Ceramic, Nanocomposite, Detoxification, Decolorization

## Abstract

This research paper details the creation of innovative nanocomposites using the sol-gel technique, incorporating polyoxometalates SiW_9_Ba_3_ to stabilize ceramic particles of strontium ferrite (SrFe_12_O_19_) polymer and Chitosan (CS). The identification and confirmation of the nanocomposites obtained at each stage were carried out through the use of FT-IR, EDX, XRD, and FESEM analyses. To evaluate their ability to remove organic dyes, we analyzed the catalytic activity of these nanocomposites during photocatalytic detoxification procedures. With its exceptional photocatalytic properties, the nanocomposite (SiW_9_Ba_3_@SrFe_12_O_19_@Cs) was able to remove estamipride poison at an impressive rate of 85 % and xylene dye solution at an even higher rate of 98 %. In addition, an extensive examination was undertaken to explore the primary variables that influence process efficiency. The study suggests that ceramic nanocomposites incorporating heteropolyoxometalate may offer a viable approach to effectively eradicate pollutants from the environment.

## Highlights

• The heterogeneous nanocatalyst (SiW_9_Ba_3_@SrFe_12_O_19_@CS) was synthesized successfully.
• The catalytic activity of SiW_9_Ba_3_@SrFe_12_O_19_@CS was tested on the removal of environmental pollutants.
• SiW_9_Ba_3_@SrFe_12_O_19_@CS remove 85 % of estamipride and 98 % of xylene dye solution.

## 1 Introduction

The world is currently grappling with a problem known as pollution, caused by the progress of human civilization, technological advancements, and the growing population. This poses a threat to the lives of all living beings on the planet. Statesmen from all countries prioritize environmental protection, recognizing its significance. In today's world, pollution has reached a point where the well-being of people in a city or even a country can be jeopardized by the environmental conditions of another city or country [[1], [2], [3]]. To illustrate, the use of pesticides has the potential to contaminate the environment, impacting essential components like water, air, and soil, much like agricultural pesticides. If colored wastewaters produced by industries like paper making, textile, rubber, and plastic manufacturing are released into the environment without treatment, they can pose numerous and significant environmental issues. Among its various impacts, the discharge of this wastewater into receiving waters can result in ecosystem toxicity and the potential for bioaccumulation in organisms [[4], [5], [6]]. Various techniques, including membrane filtration, flocculation, adsorption, ozonation, photodegradation, and biodegradation, have been employed to treat dye-containing wastewater. However, each of these methods has its own set of limitations. The advanced oxidation process (AOP), like the Fenton reaction, has been shown to successfully break down dangerous pollutants using hydrogen peroxide (H_2_O_2_). Despite its benefits, the long-term application of this method is limited due to the challenges of storing H_2_O_2_, maintaining extreme operating pH, and dealing with the production of iron sludge [[7], [8], [9]]. Polyoxometalates (POMs), which are a vast group of environmentally friendly inorganic compounds, have a profound impact on the realms of science and technology due to their diverse structure and wide range of applications [10,11]. POMs have found diverse applications in different fields, such as optics, magnetism, catalysis, biology, and electronics [[12], [13], [14]]. These successes can be attributed to the unique properties of these materials, including their diverse sizes, intricate structures, impressive redox capabilities, strong ion charges, and substantial weight. Apart from the mentioned fields, POMs have found widespread application in pharmaceuticals, medicine, analytical chemistry, dye and pigment industry, sensors, and gas absorbers. Additionally, they are utilized in membranes and separators, electrochemistry, cation exchange resins, and food chemistry [15]. These materials possess remarkable absorbent properties and are frequently employed to address environmental issues, including the elimination of harmful pollutants from water solutions. POMs have the capacity to interact with and stabilize proteins, and this ability is influenced by their charge and size [16]. In addition, POMs exhibit comparable hierarchical structural characteristics and perform functions akin to enzymes, which are nature's biological catalysts. The efficient catalysis of reactions by POMs, functioning as enzymes, is largely dependent on their structure and redox activity. Nonetheless, in aqueous solutions, their activities are fleeting and subject to changes in pH [17,18]. Khan et al. synthesized two new compounds in their study: a sandwich-type covalently attached fluorinated anhydride called [C_27_H_24_F_6_N_2_O_10_](FDA-Di-Tris) and a Dawson POM hybrid known as [{N(C_4_H_9_)_4_}_10_ {C_22_H_16_F_6_N_2_O_128_P_4_V_6_W_30_·CH_3_CN}](FDA@Di-POM). They observed drug loading efficiencies of 82 % for FDA@Di-POM and 57 % for FDA-Di-Tris. In basic media (pH = 11), the drug release for FDA-Di-Tris was observed to be the lowest at 14 %, whereas in acidic media (pH = 3), it was the highest at 59 %. Similarly, FDA@Di-POM showed similar behavior in both media, with a release of approximately 55 % in basic media (pH = 11) and 49 % in acidic media (pH = 3) [19]. According to Zeb et al. (2018), the synthesized iron-based POMs have the ability to remove rhodamine B dye by undergoing a Photo-Fenton reaction, which is a type of advanced oxidation process (AOP). Despite this, the research on POMs still heavily revolves around photo-based reactions, with minimal efforts dedicated to investigating their potential use in non-photo reactions, such as catalyst activated AOP [20]. For instance, as the pH gradually increases, the silicotungstate Keggin ion, SiW_12_, undergoes a series of structural transformations [21,22]. The interconversions result in modifications to the structural composition of the POM, leading to alterations in its reactivity. The mono-lacunary Keggin ion, {SiW_11_}, exhibits higher reactivity compared to {SiW_12_} and demonstrates the ability to coordinate metal ions7 or undergo reactions with electrophiles such as phosphonates or silicates [22]. The speciation of multiple POMs with pH was thoroughly examined in a recent review by Rompel and co-workers, underscoring the crucial role of these equilibria in maintaining POM stability. Interested readers are encouraged to use this article as a Rosetta stone for understanding the true nature of the POM present in their system [23,24]. In addition, POMs possess robust Brønsted acidity, enabling them to transfer protons in solution as well as in the solid state, resulting in the formation of stable anionic clusters [25]. The paper delves into the examination of different techniques for detoxifying and eliminating organic dyes, along with their wide-ranging uses and focuses on designing and synthesizing ceramic nanocomposites using Keggin-type polyoxometalates as the base material. In this work, to enhance the advantages of heterogeneous catalysts, such as recovery and separation capability, and to boost the catalytic activity of polyoxometalates, a new Keggin-type species (SiW_9_Ba_3_) was synthesized and stabilized on the substrate SrFe_12_O_19_ and chitosan, using the sol-gel method. Identification of the SiW_9_Ba@SrFe_12_O_19_@CS catalysts involved comprehensive analysis techniques such as MAP, SEM and XRD, UV–vis, and FT-IR. These nanocomposites were tested for their catalytic performance in the decolorization of xylene and the detoxification of acetone. Additionally, the study examined the impact of key factors, including catalyst quantity, temperature, time, and catalyst type, on process efficiency.

## 2 Experimental

### 2.1 Materials

Commercially accessible materials and solvents are readily used. The following chemicals were obtained from Sigma-Aldrich: Iron (III) nitrate nonahydrate (Fe(NO_3_)_3_.9H_2_O) with the purity of more than 99.95 %, Strontium (II) nitrate (Sr(NO_3_)_2_.4H_2_O) with the purity of more than 99 %, Nitric acid (HNO_3_) with the purity of more than 99 %, Barium acetate (C_4_H_6_BaO_4._4H_2_O) with the purity of more than 98 %, Sodium bicarbonate (NaHCO_3_) with the purity of more than 30 %, ethanol (C_2_H_5_OH) with the purity of more than 99.8 %, acetic acid (CH_3_COOH) with the purity of more than 99 %, silicotungstic acid (H_4_SiW_12_O_40_._n_H_2_O) with the purity of more than 95 %, Chitosan (C_6_H_11_NO_4_) with the purity of more than 99 %; and the following chemicals were obtained from Merck: Citric Acid Monohydrate (C_6_H_8_O_7_. H_2_O), Hydrochloric acid (HCl) with the purity of more than 37 %, sodium hydroxide (NaOH) with the purity of more than 99 %, Potassium chloride (KCl), Methyl orange (C₁₄H₁₄N₃NaO₃S) with the purity of more than 99.99 %, and Xylenol Orange tetrasodium (C_31_H_28_N_2_Na_4_O_13_S).

### 2.2 Characterizations methods

Fourier transform infrared (FT-IR) spectroscopy was conducted using KBr pellets using Thermo scientific SMART OMNI TRANSMISSIO Nicolt- Is10. The spectra were collected over a range of 400–4000 cm^−1^. Ultra violet and visible spectrophotometry, Shimadzu 160 spectrometer, has been used using absorption wavelengths to show the type of graft in the studied species and the measurements were taken within the wavelength range of 190–790 nm. Czechia scanning electron microscope was used to examine the SiW_9_Ba@SrFe_12_O_19_@CS nanoparticles to obtain FE-SEM images. Additionally, elemental mapping analysis was performed to visualize the distribution of different elements within the samples. X-ray diffraction (XRD) was used to study the crystallinity of prepared nanomaterials, with Cu Kα radiation (λ = 1.5406 Å) for powder XRD, using X'Pert pro Philips. Using a andelin Sonorex Digitec frequency 35 kHz mains connection 230 V Ultrasonic, the solution becomes homogenous, and also by creating strong pressure waves in a liquid environment, it causes flow in the liquid and under suitable conditions causes cavitation phenomenon. The bursting of the bubbles produces a shock wave with enough energy to break the covalent bond.

### 2.3 Synthesis of strontium hexaferrite (SrFe_12_O_19_)

**Scheme 1:**
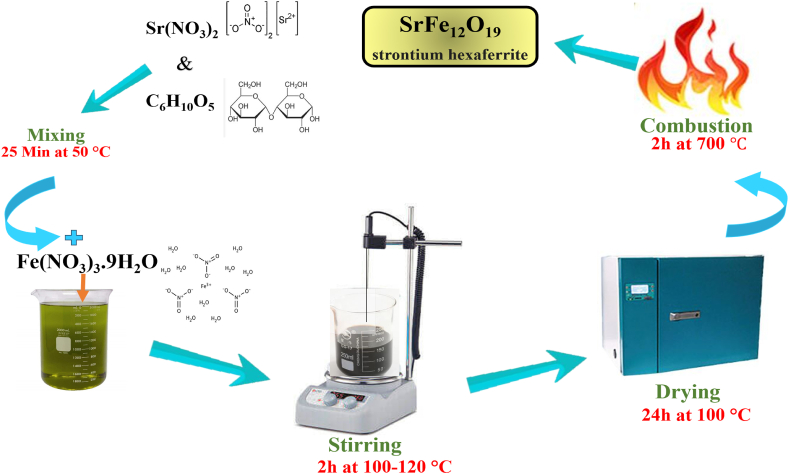
Synthesis of strontium hexaferrite (SrFe_12_O_19_).

By employing the sol-gel method, researchers successfully synthesized strontium hexaferrite. The first step involved dissolving the appropriate quantities of strontium nitrate (0.0634 g) and the reducing agent (0.632 g) sourced from starch in 25 ml of distilled water. Strontium nitrate was mixed with a starch solution and the resulting mixture was then subjected to a temperature of 50 °C for 25 min. A quantity of iron nitrate weighing 1.454 g was dissolved in 25 ml of distilled water and combined with the previous solution. The resulting mixture was then heated at a temperature range of 100–120 °C for duration of 2 h. At this point, a thick brown gel was acquired, which was subsequently dehydrated in an oven set at 100 °C for duration of 24 h. Eventually, it underwent a calcination process at a temperature of 700 °C for duration of 2 h (Scheme 1).

### 2.4 Synthesis of Keggin-type polyoxometalate with incomplete three-substituted structure (SiW_9_Br_3_)

**Scheme 2:**
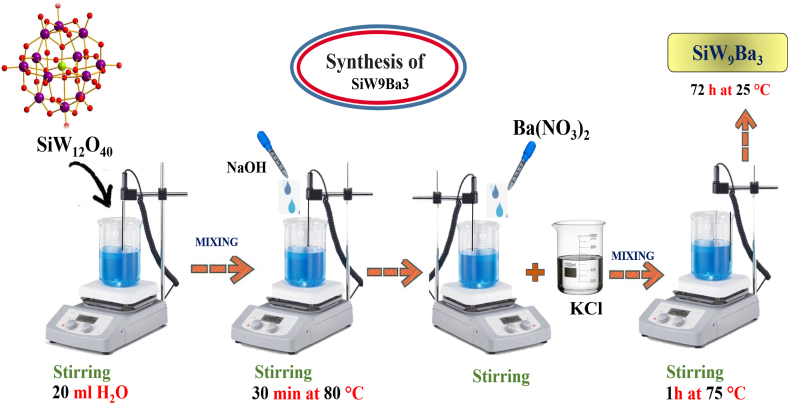
Synthesis of Keggin-type polyoxometalate with incomplete three-substituted structure.

In order to accomplish this, we dissolved 0.60 g of SiW_12_O_40_ in 20 ml of distilled water, and then we placed the solution on the stir plate and stirred it well. Following the cleaning and calibration of the pH meter, the solution was carefully poured into the beaker and the initial pH level was documented. The stirring solution was treated with a dropwise addition of 0.1 M NaOH solution until the pH level reached 6.50. The solution described above was heated to a temperature of 80 °C and kept at that temperature for 30 min. A solution of barium nitrate (0.05 g) was prepared by dissolving it in a minimal amount of distilled water. This solution was then carefully added drop by drop to replace the cavity with barium. The resulting solution received an addition of 10 mL of saturated potassium chloride solution (KCl). Additionally, the solution was agitated at a temperature of 75 °C for approximately 1 h. After obtaining the solution, we allowed it to settle at ambient temperature for 72 h until precipitation was fully achieved (Scheme 2).

### 2.5 Synthesis of nano catalysts SiW_9_Ba_3_@SrFe_12_O_19_@CS

**Scheme 3:**
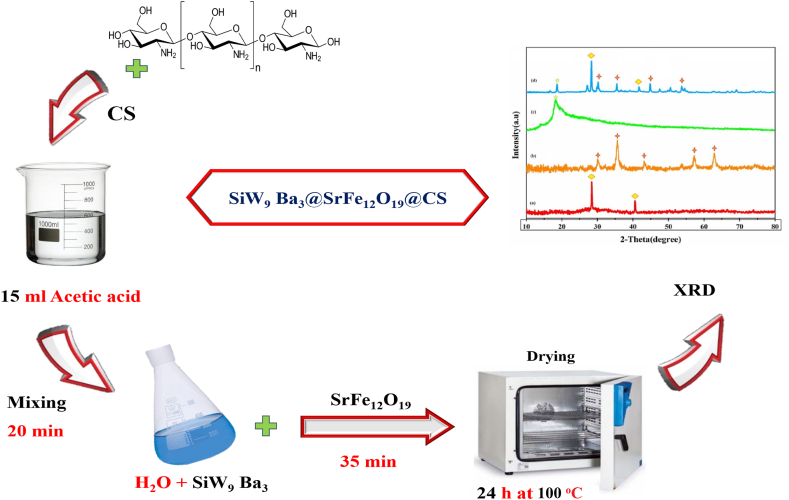
Synthesis of nano photocatalysts SiW_9_Ba_3_@SrFe_12_O_19_@CS

Initially, 0.28 g of CS chitosan was combined with 15 ml of 2 % acetic acid at room temperature to ensure complete homogenization. Subsequently, a small amount of distilled water was used to dissolve 0.10 g of the synthesized polyoxometalates, which was then slowly added to the aforementioned solution over duration of 20 min. Within a time frame of 35 min, the above solution was supplemented with 0.07 g of strontium hexaferrite (SrFe_12_O_19_) in the next step. To dry the final product solution (attained gel), it was placed in an oven at 100 °C overnight (Scheme 3) [[27], [28], [29]].

### 2.6 Decolorization process with SiW_9_Ba_3_@SrFe_12_O_19_@CS nanocomposite

Magnetic stirring was used in 80 mL glass containers for all decolorization experiments. A certain concentration of xylene orange dye solutions was prepared using a volume of 20 mL. Distilled water was used to dilute the dye solutions as well. A particular quantity of nano-absorbent SiW_9_Ba_3_@SrFe_12_O_19_@CS was introduced into the solutions and agitated at ambient temperature for a specified duration. Once the specified time elapsed, 5 mL samples were extracted using a syringe and subsequently placed in a centrifuge, operating at a speed of 2000 rpm for a duration of 15 min. The spectrophotometer was used to measure the absorption of specific concentrations. Equation (1) was used to calculate the color concentration at various stages, and equation (2) was used to determine the percentage of color removed.

(1) $$M_{1}V_{1}=M_{2}V_{2}$$

(2) $$Dye\;removal\;officiency\left(\%\right)=\frac{M_{0}-M}{M_{0}}\times 100$$

### 2.7 Check removal in optimal values

Initially, 20 ml of xylene orange solution was added to an 80 ml beaker, followed by the addition of 0.005 g of SiW_9_Ba_3_@SrFe_12_O_19_@CS. The mixture was vigorously stirred to ensure uniformity, and then exposed to UV light for 60 min. Subsequently, 5 ml of dye was added to a test tube and transferred to a centrifuge, where it was spun for 15 min. The decrease in radiation energy of organic compounds results in a shift in their interaction with chemical compounds, making them more prone to chemical oxidation. Additionally, radiation can effectively generate hydroxyl radicals by interacting with water. These radicals have a stronger oxidizing potential compared to water, making them highly efficient in oxidizing wastewater constituents. In addition, SiW_9_Ba_3_@SrFe_12_O_19_@CS was exposed to different lighting conditions, including darkness, visible light, and ultraviolet light. Additionally, the study examined the percentage of removal through analysis of various factors such as time, temperature, heat, color mixture, and color concentration. The optimal values obtained from this investigation were then further explored in the third chapter.(see Scheme. 4)

**Schem. 4:**
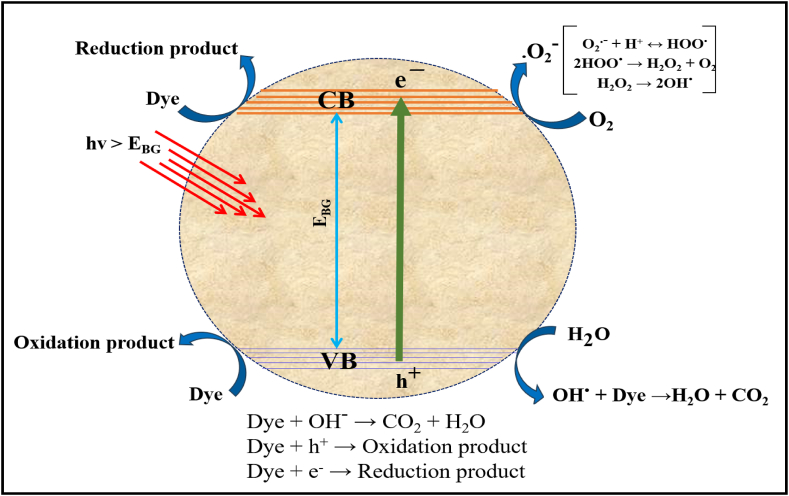
Mechanism of the photocatalytic SiW_9_Ba_3_@SrFe_12_O_19_@CS reactions.

### 2.8 Detoxification process with SiW_9_Ba_3_@SrFe_12_O_19_@CS nanocomposite

Acetami Prid is the poisonous substance utilized in this project. Additionally, human participants underwent detoxification experiments with 80 mL while being stirred using a magnetic method. Solutions containing a specific concentration of a toxic substance were prepared, with a volume of 20 mL. Additionally, distilled water was employed for diluting poisonous solutions. We added a precise quantity of SiW_9_Ba_3_@SrFe_12_O_19_@CS to the solutions and mixed them at ambient temperature for a designated duration.

Once the designated time had passed, 5 mL samples were extracted using a syringe and then placed in a centrifuge, spinning at a speed of 2000 rpm for a duration of 15 min. To measure absorption, a spectrophotometer was used with specific concentrations. The sample SiW_9_Ba_3_@SrFe_12_O_19_@CS was subjected to varying light conditions including dark, visible, and ultraviolet. Additionally, the percentage of removal was analyzed by considering factors such as time, temperature, heat, poison mixture, and poison concentration, resulting in the identification of optimal values.

## 3 Results and discussion

### 3.1 Characterization of SiW_9_Ba_3_@SrFe_12_O_19_@CS nanocatalyst

#### 3.1.1 FT-IR spectra

**Fig. 1:**
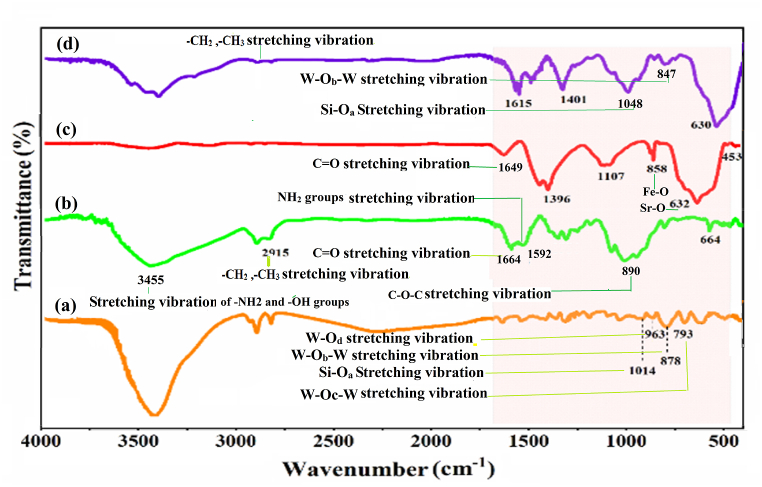
IR spectrum of SrFe_12_O_19_ (a), (b) CS, (c) SiW_9_Ba_3_ and (d) SiW_9_Ba_3_@SrFe_12_O_19_@CS.

The FT-IR spectroscopy was employed to verify the successful synthesis of the as-prepared nanocomposite SiW_9_Ba_3_@SrFe_12_O_19_@CS. Fig. 1 displays the infrared spectra of (a) SiW_9_Ba_3_, (b) CS, (c) SrFe_12_O_19_, and (b) the nanocatalyst SiW_9_Ba_3_@SrFe_12_O_19_@CS. FT-IR spectrum of Keggin-type polyoxometalate SiW_9_Ba_3_ (a) shows four specific peaks related to Keggin-type structure: absorption peak at 1014 cm^−1^ related to stretching vibration (Si-O_a_), 963 cm^−1^ peak related to double bond of oxygen with metal (W-O_d_), the peak of area 878 cm^−1^ is related to oxygen in the shared corner between two octahedra (M-O_b_-M) and the absorption peak 793 cm^−1^ is related to oxygen that is bridged (M-Oc-M) between two octahedral the common edge is placed [[26], [27], [28], [29]]. Additionally, when examining the chitosan (CS) spectrum (b), the peaks in the 2915 cm^−1^ region correspond to the stretching vibration of C–H, while the peaks in the 1664 and 1592 cm^−1^ region are attributed to C=O and N–H. In FT-IR spectrum of SrFe_12_O_19_ (c), peaks attributed to O–H bending vibrations are observed in the 1622 cm^−1^ and the peak of area 858 cm^−1^ is related to stretching vibration Fe–O and the absorption peak 632 cm^−1^ is related to stretching vibration Sr–O. Fig. 1 (d) shows the FT-IR spectrum of the SiW_9_Ba_3_@SrFe_12_O_19_@CS nanocomposite, where the peaks corresponding to Keggin-type's polyoxometalate, CS and SrFe_12_O_19_ are clearly visible. This indicates successful synthesis of the mentioned nanocomposite.

#### 3.1.2 XRD patterns

**Fig. 2:**
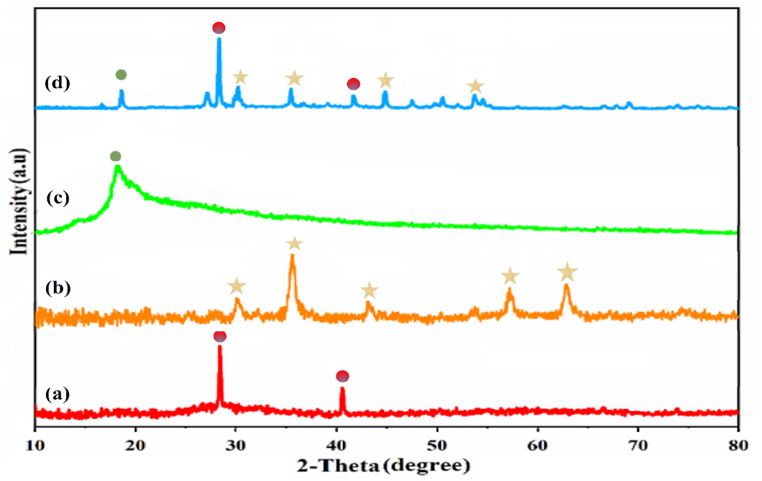
XRD pattern of (a) SiW_9_Ba_3_, (b) SrFe_12_O_19_, (c) CS and (d) SiW_9_Ba_3_@SrFe_12_O_19_@CS.

The XRD patterns of SiW_9_Ba_3_, SrFe_12_O_19_, CS, and SiW_9_Ba_3_@SrFe_12_O_19_@CS compounds were analyzed to investigate their crystal structures. In Fig. 2 (a) the XRD patteren of SiW_9_Ba_3,_ peaks at 28.41^o^ and 40.57^o^ are clearly visible. Various typical peaks at 2θ ∼ 28 and 41° display the XRD patterns of the Keggin-type POM (SiW_9_Ba_3_). The XRD spectrum in Fig. 2 (b) of SrFe_12_O_19_ displays distinct and sharp peaks at 2θ values of 30.107°, 35.51°, 43.16°, 53.52°, 57.01°, and 62.75°. These peaks were compared to the reference card (JCPDS No. 24–1207) [9,[30], [31], [32]]. The pattern of chitosan in Fig. 2 (c) indicates its amorphous nature, as evidenced by a broad peak and a peak at 19.83°, which suggests its semi-crystalline characteristics. In Fig. 2 (d), the SiW_9_Ba_3_@SrFe_12_O_19_@CS nanocomposite pattern displayed the peaks of CS and SrFe_12_O_19_ substrate, albeit slightly shifted. Additionally, several polyoxometalate peaks were detected in the synthesized nanocomposite, indicating a homogeneous distribution of polyoxometalate particles on the substrate. Additionally, it was approximated that the particles had a size of approximately 33 nm based on the Debye−Scherrer equation (eq (3)),54 where D is the nanocrystallite size (nm), λ is the X-ray wavelength (Cu Kα radiation: λ = 0.1540 nm), fwhm is the full width at half maximum in radians, and θ is the Bragg angle.

(3) $$D=\frac{k\lambda }{\text{fwhm}\;\cos\;\theta }$$

#### 3.1.3 SEM analysis and results

**Fig. 3:**
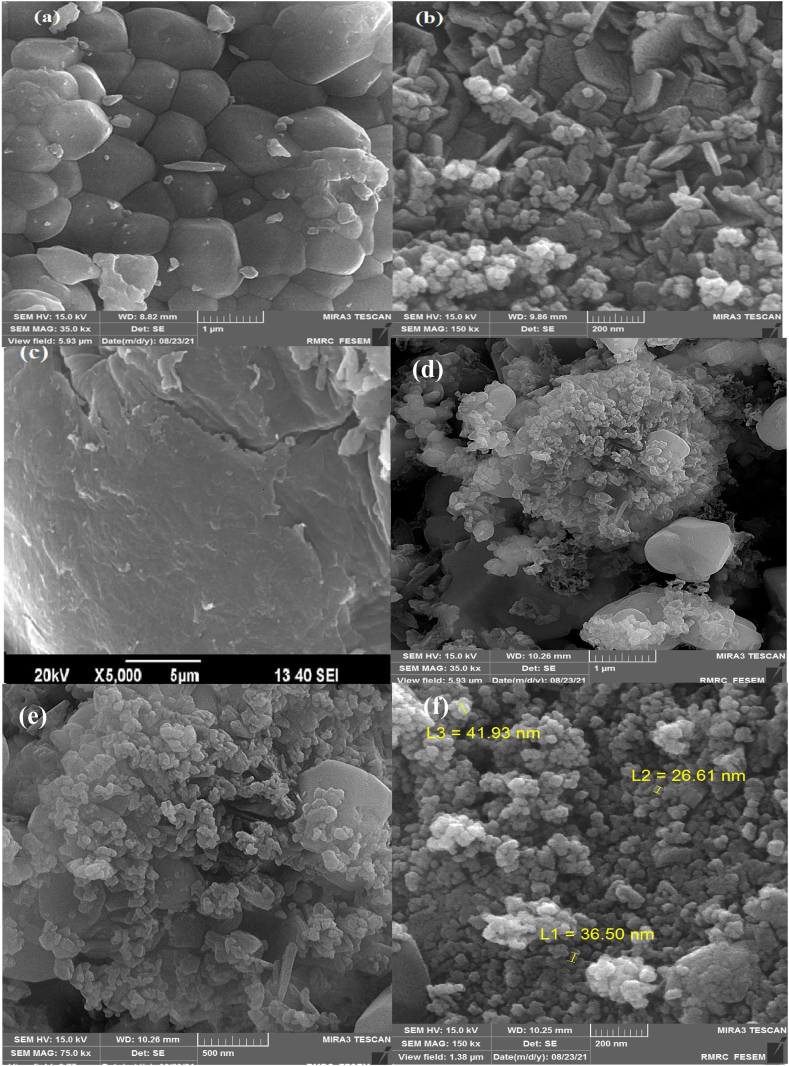
SEM image of (a) SiW_9_Ba_3_, (b) SrFe_12_O_19_, (c) CS and (d,e and f) SiW_9_Ba_3_@SrFe_12_O_19_@CS.

**Fig. 4:**
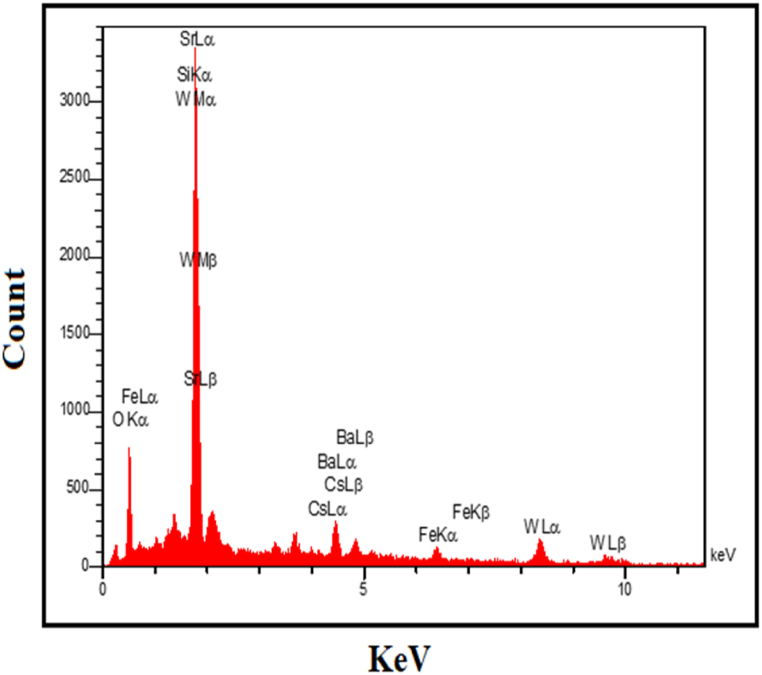
EDX micrograph of nanocomposite SiW_9_Ba_3_@SrFe_12_O_19_@CS.

Scanning electron microscopy was used to examine the morphology of the synthesized materials [33], and the results were recorded in SEM images (Fig. 3a–f). Additionally, this Fig. 3a displays agglomerate particles of SiW_9_Ba_3_ polyoxometalate and crystallinity polygonal shape which confirms the data obtained from XRD reflections. Fig. 4b, depicts the polygonal and dense agglomerated shape of SrFe_12_O_19_ with a good surface-to-volume ratio index which makes it a remarkable candidate for hosting another crystal shape compound like SiW_9_Ba_3_. Furthermore, upon comparing the SEM images of the raw materials with the nanocomposite (Fig. 3d–f), it is evident that the morphology of the nanocomposite particles closely resembles that of the substrate. Additionally, the polyoxometalate particles are evenly and uniformly distributed across the substrate's surface.

#### 3.1.4 EDX and map analysis and results

**Fig. 5:**
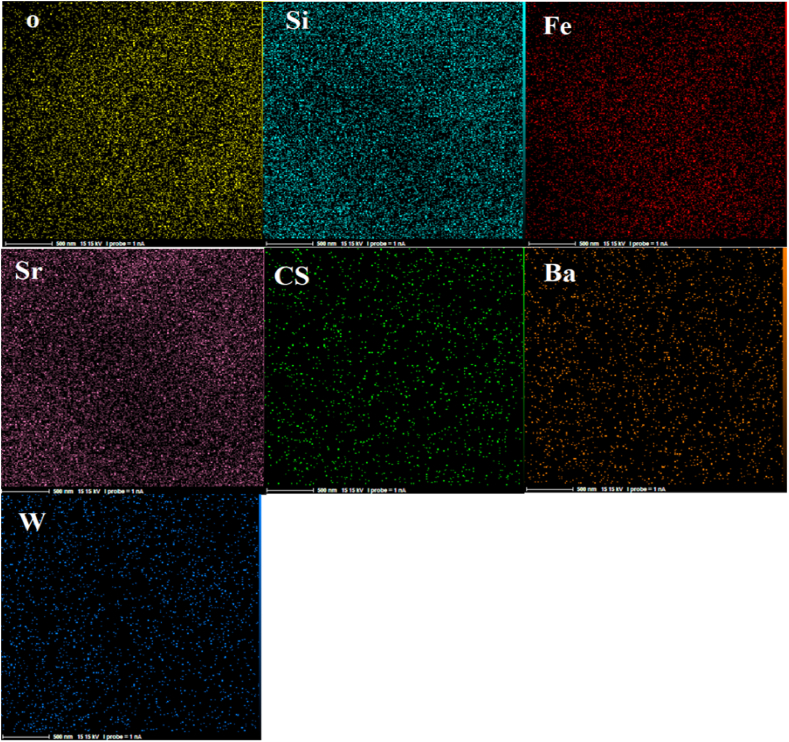
Scattering images of elements related to SiW_9_Ba_3_@SrFe_12_O_19_@CS nanocatalyst.

By examining the EDX micrograph displayed in Fig. 4, it is evident that the SiW_9_Ba_3_@SrFe_12_O_19_@CS nanocomposite contains Sr, O, W, Fe, Ba, Si, and CS elements, each with distinct weight percentages. The uniform preparation and distribution of elements in the nanocomposite can also be observed in the images of Fig. 5 Map, indicating the successful synthesis of the SiW_9_Ba_3_@SrFe_12_O_19_@CS catalyst.

The catalytic activity of the nanocomposite SiW_9_Ba_3_@SrFe_12_O_19_@CS was investigated to determine its effectiveness in decolorizing xylene orange. The decolorization process was carried out using ultraviolet, dark, and visible light to study the photocatalytic property of SiW_9_Ba_3_@SrFe_12_O_19_@CS.

The study focused on investigating the decolorization process under UV light, which showed superior performance compared to other methods. The research also examined the influence of different factors, such as the amount of catalyst, color concentration, temperature, and color mixture.

### 3.2 Xylene orange decolorization under visible, ultraviolet and dark radiation

To demonstrate the ability of xylene orange solution to remove color, it was subjected to visible, ultraviolet, and dark light.

**Fig. 6:**
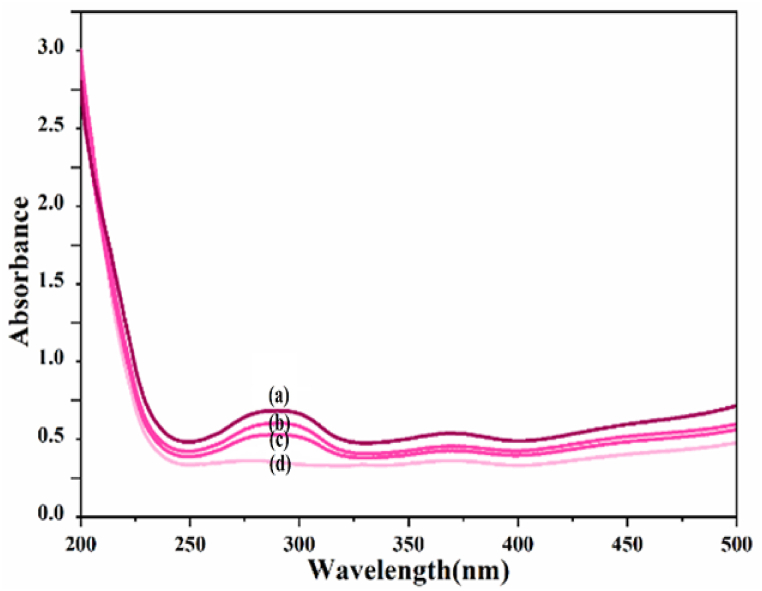
UV–vis spectrum of xylene orange solution under (a) zero radiation, (b) darkness, (c) Visible, (d) Ultraviolet. (For interpretation of the references to color in this figure legend, the reader is referred to the Web version of this article.)

The greatest percentage of color removal was observed under ultraviolet light. To confirm the ongoing experiment, ultraviolet light will be employed, as depicted in Fig. 6.

### 3.3 Investigating the effect of SiW_9_Ba_3_@SrFe_12_O_19_@CS photocatalyst on decolorization under UV radiation

**Fig. 7:**
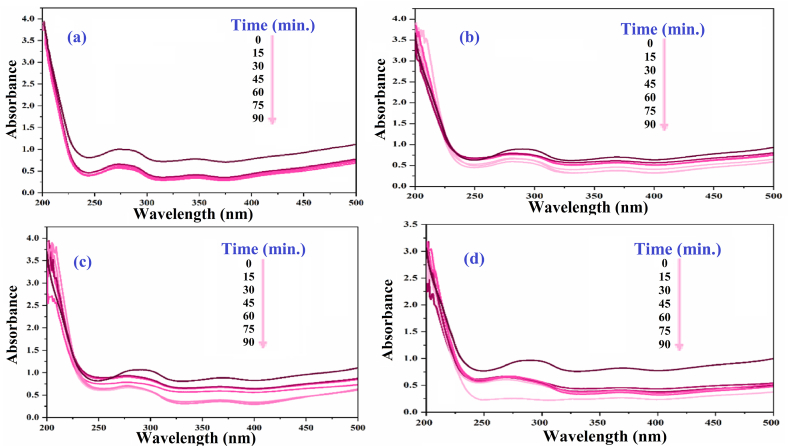
UV–vis spectrum of xylene orange dye solution after decolorization in the presence of a) 0.005 g, b) 0.01 g, c) 0.02 g, and d) 0.03 g. (For interpretation of the references to color in this figure legend, the reader is referred to the Web version of this article.)

In this study, we examined the impact of different amounts of photocatalyst (0.005g, 0.01g, 0.02g, and 0.003g) on the decolorization of a 30 ml solution containing 15 ppm xylene orange. According to Fig. 7, the most effective removal of xylene orange was achieved when using a g value of 0.03 with SiW_9_Ba_3_@SrFe_12_O_19_@CS. Additionally, the amount of removal did not change even after adding more catalyst. Thus, by removing 99 % of the color from the photocatalyst, we achieved an optimal g value of 0.03. Fig. 7a-d illustrates how the removal of xylene orange color is affected by increasing the amount of photocatalyst. Increasing the quantity of photocatalyst leads to faster degradation of xylene orange due to the larger absorber surface area and improved accessibility to absorption sites.

### 3.4 Investigating the effect of concentration

**Fig. 8:**
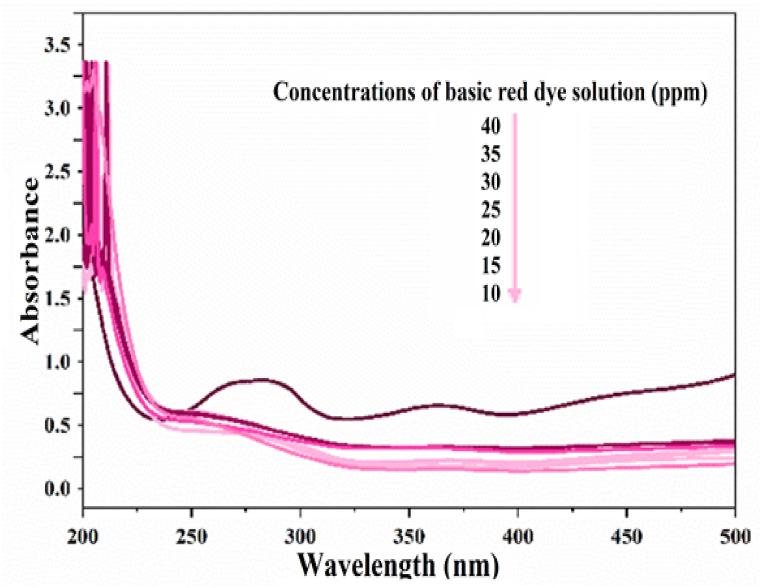
UV–vis spectrum of basic red dye solution after decolorization in concentrations of 10–40 ppm by photocatalyst SiW_9_Ba_3_@SrFe_12_O_19_@CS. (For interpretation of the references to color in this figure legend, the reader is referred to the Web version of this article.)

The process of removing color from a xylene orange solution was conducted using various concentrations (10, 15, 20, 25, 30, 35, 40 ppm) and with the addition of 0.03 g of photocatalyst SiW_9_Ba_3_@SrFe_12_O_19_@CS. The decolorization process was carried out under UV radiation for a duration of 90 min. At a concentration of 10 ppm, the color removal percentage reached 99 % within 90 min. The concentration of 40 ppm achieves a color removal percentage of 34.2 % within the specified time frame. With an increase in color concentration, it is evident that the percentage of color removal decreases. This is attributed to the time required for color molecules to reach the catalyst surface and undergo destruction. Additionally, when the color concentration increases, some of the light is absorbed by the color instead of reaching the catalyst surface for activation (Fig. 8).

### 3.5 Decolorizing brush of basic red and methyl orange mixture

**Fig. 9:**
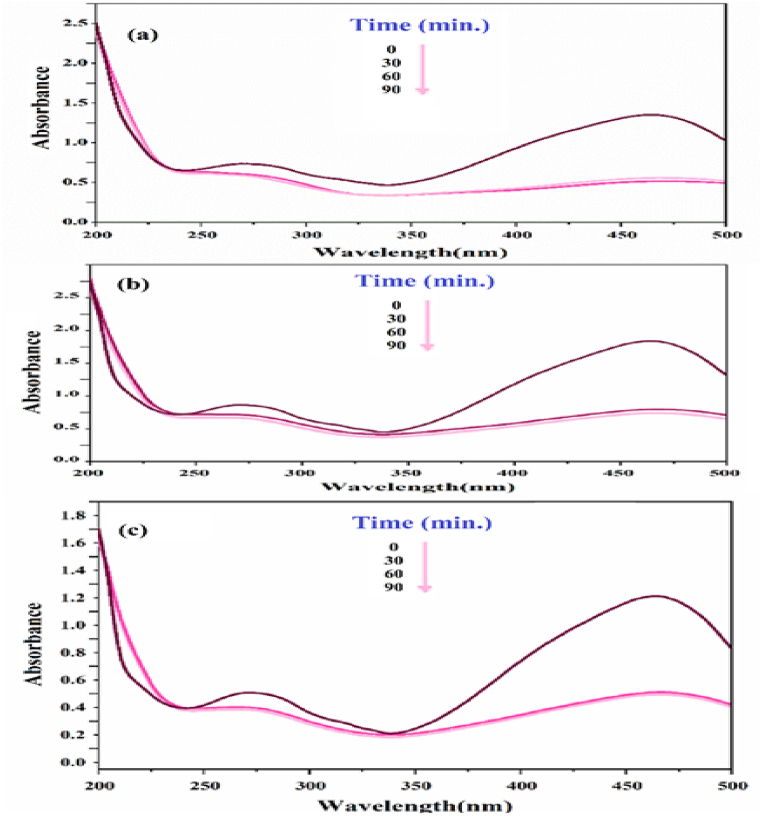
decolorization of mixed solution of xylene orange and methyl orange in the presence of 0.03 g of SiW_9_Ba_3_@SrFe_12_O_19_@CS nanocomposite under UV light irradiation: xylene orange 30 ppm and methyl orange a) 5 ppm, b) 10 ppm, and c) 15 ppm. (For interpretation of the references to color in this figure legend, the reader is referred to the Web version of this article.)

In the present time, wastewater can have various colors. During this experiment, we examined the effects of mixing solutions with two different colors and testing them for decolorization. Xylene orange and methyl orange were combined, followed by the addition of 0.03 g of photocatalyst. The solution was then subjected to ultraviolet light exposure. Fig. 9(a–c) illustrates the displayed results.

### 3.6 Examination of temperature effect

**Fig. 10:**
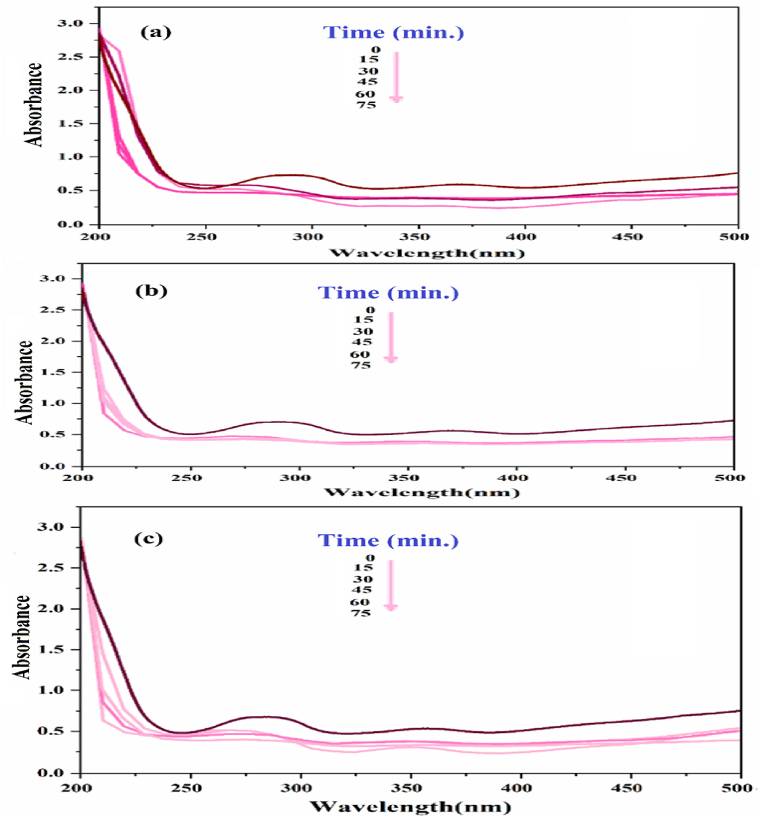
Effect of temperature in photocatalytic process (a) 27 °C, b) 45 °C, and c) 60 °C.

Based on the test procedure and the obtained results, it is evident that temperature directly impacts the removal of the coloring solution. The data presented in Fig. 10a-c indicates that there is a direct relationship between temperature and color degradation, with higher temperatures leading to greater degradation. It was determined that the best temperature was 60 °C.

### 3.7 Investigating the photocatalytic activity of SiW_9_Ba_3_@SrFe_12_O_19_@CS nanocomposite in the detoxification process of osmosis

In order to conduct the photocatalytic test on the estamipyrid toxin solution, we assessed multiple variables, including catalyst concentration, toxin concentration, and the duration of the detoxification reaction under ultraviolet light radiation.

#### 3.7.1 Examining the effective conditions on the detoxification of osmosis under visible, dark and ultraviolet radiation

**Fig. 11:**
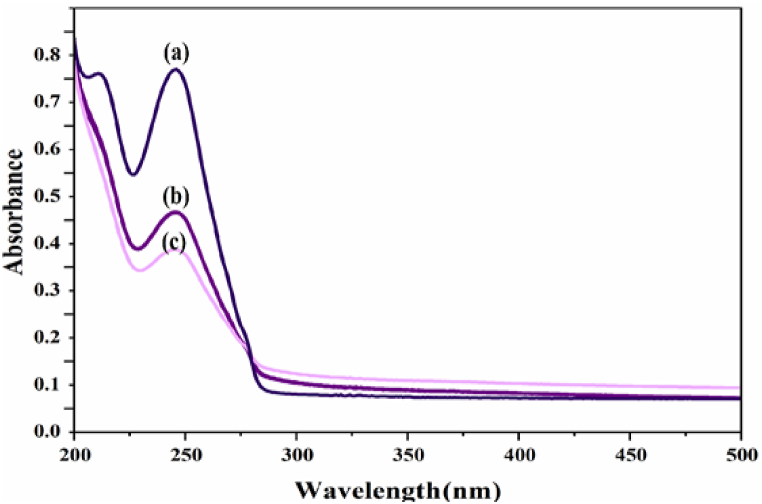
UV–vis spectrum of bromide toxic solution under (a) dark, (b) visible and (c) ultraviolet radiation.

The initial examination of the toxic solution with a photocatalyst was conducted using visible, ultraviolet, and dark light (Fig. 11). The most effective method for removing toxins was found to be ultraviolet light, resulting in the highest percentage of removal. UV light will be used to verify the progress of the experiment.

#### 3.7.2 Investigating the effect of catalyst amount

**Fig. 12:**
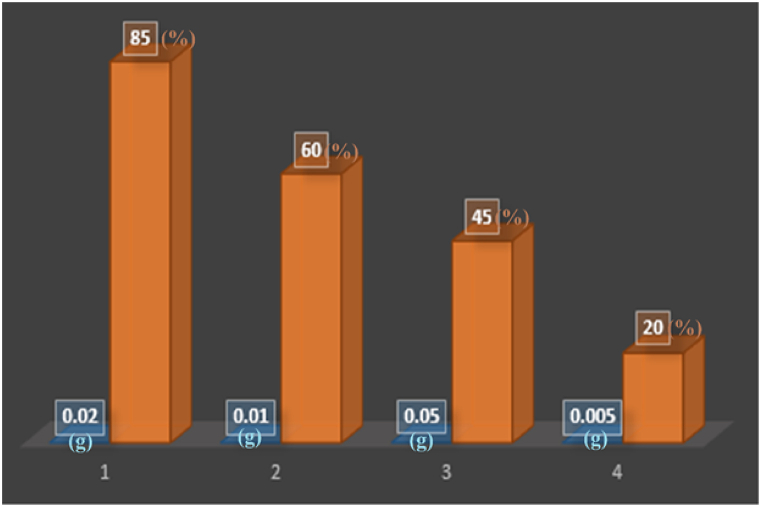
different concentrations of the synthesized SiW_9_Ba_3_@SrFe_12_O_19_@CS catalyst on the removal percentage of bromine poison.

Fig. 12 illustrates the investigation into the influence of increasing the SiW_9_Ba_3_@SrFe_12_O_19_@CS catalyst concentration on the efficient elimination of toxins. The study revealed that as time and catalyst concentration increased, there was a corresponding increase in the percentage of toxin removal. The optimal concentration for toxin removal was determined to be 0.02 g.

#### 3.7.3 Effect of toxin concentration

**Fig. 13:**
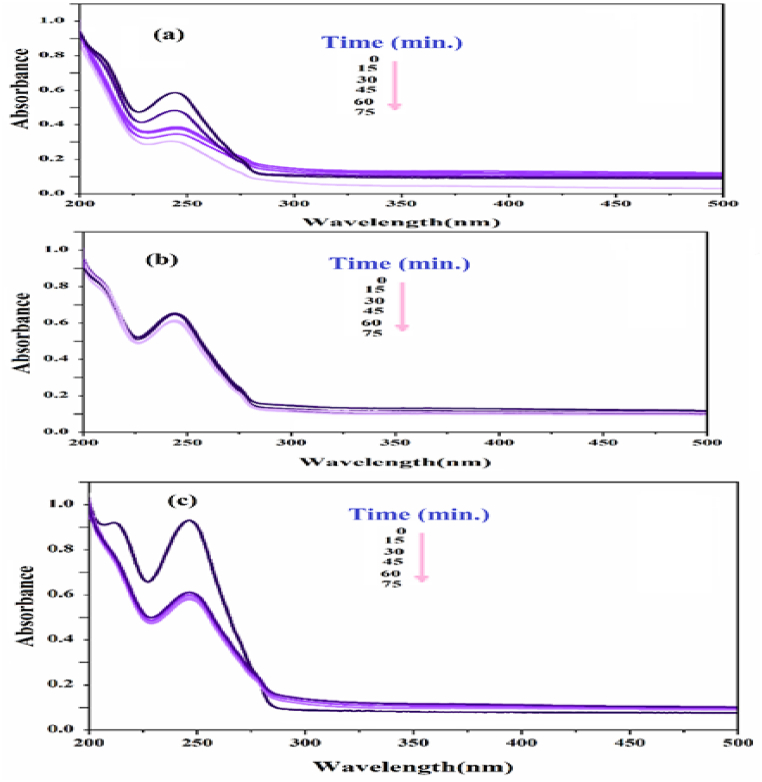
UV–visible spectrum related to a) 10 ppm, b) 20 ppm, and c) 30 ppm poison concentration at different times in the presence of the synthesized SiW_9_Ba_3_@SrFe_12_O_19_@CS catalyst.

The detoxification of photocatalyst was studied in comparative experiments using different concentrations (10, 20, and 30 ppm) of acetamine bromide under ultraviolet light. The findings are presented in Fig. 13a-c.

### 3.8 Mechanism of the photocatalytic reactions

By utilizing UV light sources, researchers can delve into the light harvesting capability of nanocatalysts that depend on SiW_9_Ba_3_@SrFe_12_O_19_@CS structures. Due to its internal electrical structure, the valence band (VB) experiences the slow excitation of photo-induced electrons (e), which in turn generates accumulated holes (h*) in the conduction band (CB). The catalytic process heavily relies on the presence of electron-hole pairs, which are primarily generated by the binary semiconductor, especially under high light intensity. Due to the internal electrical structure, the movement of photo-induced electrons from the valence band to the conduction band creates accumulated holes (h*) in the valence band (equations (4), (5), (6), (7), (8), (9), (10), (11), (12), (13), (14), (15), (16), (17), (18), (19), (20), (21))). In one particular process, O2 molecules dissolved on the catalyst surface effortlessly capture carrier transport e ¯, leading to the creation of O2 radicals. On the other hand, the active vacancies provide an environment where hydroxyl molecules or adsorbed H_2_O can directly interact, leading to the production of OH radicals. The oxidation of organic dyes occurs as a direct consequence of the photodegradation process. The oxidation and reduction reactions observed in photocatalytic experiments can be significantly impacted by the presence of active species such as O_2_, OH•, and h* radicals, along with peroxide radicals. The trapping experiments aimed to gain a deeper understanding of the photocatalytic degradation process in the SiW_9_Ba_3_@SrFe_12_O_19_@CS binary nanostructure [[34], [35], [36]], focusing on the mechanisms involved (equations (4), (5), (6), (7), (8), (9), (10), (11), (12), (13), (14), (15), (16), (17), (18), (19), (20), (21))).

(4) $$\text{Nano}‐\text{photocatalyst}+\text{hv}\left(\text{UV}\;\text{light}\right)\;\to \;\text{nano}‐\text{photocatalyst}\;\left({e_{\text{CB}}}^{‐}+{{\;h}_{\text{VB}}}^{+}\right)$$

(5) $$\text{nano}‐\text{photocatalyst}\;\left({h_{\text{VB}}}^{+}\right)+OH^{‐}\;\to \;\text{nano}‐\text{photocatalyst}+OH{}^{\circ}$$

(6) $${h_{\text{VB}}}^{+}+{\;H}_{2}O\to {\;H}^{+}+OH{}^{\circ}$$

(7) $$2\;OH{}^{\circ}\to \;H_{2}O_{2}$$

(8) $${2H}^{+}+{\;O}_{2}+2{e_{\text{CB}}}^{‐}\;\to \;H_{2}O_{2}$$

(9) $${{2h}_{\text{VB}}}^{+}+{\;2H}_{2}O\;\to \;H_{2}O_{2}+{2H\;}^{+}$$

(10) $$H_{2}O_{2}+\text{hv}\;\to \;2OH{}^{\circ}$$

(11) $$O_{2}+{e_{\text{CB}}}^{‐}\;\to {{{}^{\circ}O}_{2}}^{‐}$$

(12) $${{{}^{\circ}O}_{2}}^{‐}+{e_{\text{CB}}}^{‐}\left(+{2H}^{+}\right)\;\to \;H_{2}O_{2}$$

(13) $${{{}^{\circ}O}_{2}}^{‐}+{\;H}_{2}O_{2\;}\to \;OH{}^{\circ}+OH^{‐}+{\;O}_{2}$$

(14) $${{{}^{\circ}O}_{2}}^{‐}+H^{+}\to \;HO_{2}{}^{\circ}$$

(15) $${{{}^{\circ}O}_{2}}^{‐}+{h_{\text{VB}}}^{+}{\;\to }^{1}O_{2}$$

(16) $$\text{HOO}{}^{\circ}+{{{}^{\circ}O}_{2}}^{‐}+{\;H}^{+}\;\to \;O_{2}+H_{2}O_{2}$$

(17) $$\text{XO}+\text{hv}\;\to \;XO^{*}$$

(18) $$\text{XO}*+\text{nano}‐\text{photocatalyst}\;\to \;X{O{}^{\circ}}^{+}+\text{nano}‐\text{photocatalyst}\;\left(e^{‐}\right)$$

(19) $$\text{XO}*+O_{2}{\to \;\text{XO}+}^{1}O_{2}$$

(20) $$\text{XO}+\text{nano}‐\text{photocatalyst}\;\left(e^{‐}\right)\;\to X{O{}^{\circ}}^{‐}$$

(21) $$\text{XO}+{{\;h}_{\text{VB}}}^{+}\to \;X{O{}^{\circ}}^{+}\to \;\text{decomposition}\;\text{products}\;\left(e.g.,H_{2}O,CO_{2}\right)$$

### 3.9 Comparison of photocatalyst with other reported catalysts for the removal of methyl orange

Table 1 presents the outcomes of evaluating the effectiveness of the proposed photocatalysis in comparison to other catalysts for xylene orange removal. It is evident that the desired green photocatalyst exhibits a higher degradation rate for xylene orange color compared to other catalysts.

**Table 1:** A summary of the examination and comparison of the optical degradation performance of the nanocomposite synthesized in this work with other materials prepared in previous research.

Catalyst	pH	Highest degradation (%)	Dye concentration (ppm)	Catalyst dosage (g/mL)	Source of light/Time (min)	Ref
ZnFe_2_O_4_/SrFe_12_O_19_	7	70 %/MO	10	0.02/200	Visible light/120	[18]
SrFe_12_O_19_/ZnFe_2_O_4_	7	90 %/MB	5	0.05/50	Visible light/120	[19]
TiO_2_/SrFe_12_O_19_	7	MB/94.7 %	10	200/0.02	UV-Light/300	[20]
Bi_3_O_4_Cl/SrFe_12_O_19_	–	99.7 %/RhB	10	0.1/100	solar-simulated and xenon lamp/80	[21]
SiW_9_Ba_3_@SrFe_12_O_19_@CS	7	98 %/XO	20	0.005	UV-Light/60	This study

## 4 Conclusion

This paper discusses the synthesis and evaluation of a SiW_9_Ba_3_@SrFe_12_O_19_@CS nanocomposite. To sum up, the SiW_9_Ba_3_@SrFe_12_O_19_@CS nanocomposite was synthesized using the sol-gel method. Additionally, several peaks of polyoxometalate were detected in the synthesized nanocomposite, indicating the even distribution of polyoxometalate particles on the substrate. Furthermore, it was approximated that the particles had a size of approximately 33 nm. Furthermore, by comparing the SEM images of the raw materials with the nanocomposite, it becomes evident that the morphology of the nanocomposite particles closely resembles that of the substrate. Additionally, the polyoxometalate particles are evenly distributed and uniformly dispersed across the substrate's surface. Additionally, the optimal sample underwent the photocatalysis process using visible and UV lights. Additionally, upon comparing the SEM images of the raw materials with those of the nanocomposite, it is evident that the nanocomposite particles exhibit morphology highly similar to that of the substrate, while the polyoxometalate particles show a uniform and homogeneous dispersion on the substrate's surface. The experiment included performing the photocatalysis process on the optimal sample using UV lights. Under visible light, 84 % of Xylene orange degraded when 0.005 g SiW_9_Ba_3_@SrFe_12_O_19_@CS was present, while under UV light, 93 % degradation was observed for the same concentration of Xylene orange. Based on these results, SiW_9_Ba_3_@SrFe_12_O_19_@CS appears to be a promising choice for the water purification process.

## Data availability

Data will be made available on request.

## CRediT authorship contribution statement

**Mohammad Ali Rezvani:** Writing – review & editing, Supervision, Project administration. **Amirhossein Hemmatzadeh:** Writing – original draft, Investigation. **Mir Saeed Seyed Dorraji:** Writing – review & editing, Project administration. **Narges Nourbakhsh:** Investigation. **Ghazal Oroumi:** Writing – original draft, Investigation.

## Declaration of competing interest

The authors declare that they have no known competing financial interests or personal relationships that could have appeared to influence the work reported in this paper.

## References

[1] Oikonomou A., Giannakopoulou T., Litsardakis G. (2007). Design, fabrication and characterization of hexagonal ferrite multi-layer microwave absorber. J. Magn. Magn Mater..

[2] Tetard L., Passian A., Farahi R., Davison B.H., Lereu A.L., Thundat T. (2011). Optical and plasmonic spectroscopy with cantilever shaped materials. J. Phys. Appl. Phys..

[3] Wu M., Zhang H., Yao X., Zhang L. (2001). Microwave characterization of ferrite particles. J. Phys. Appl. Phys..

[4] Jarollahi S., Nabiyouni G., Sorinezami Z., Shabani A. (2023). Synthesis and characterization of Fe3O4/TiO2/Ag magnetic nanocomposite with enhanced photocatalytic activity for methylene blue degradation and modeling by an artificial neural network (ANN). Journal of Nanostructures.

[5] Zinatloo-Ajabshir S., Esfahani M.H., Marjerrison C.A., Greedan J., Behzad M. (2023). Enhanced electrochemical hydrogen storage performance of lanthanum zirconium oxide ceramic microstructures synthesized by a simple approach. Ceram. Int..

[6] Zinatloo-Ajabshir S., Salavati-Niasari M. (2019). Preparation of magnetically retrievable CoFe2O4@ SiO2@ Dy2Ce2O7 nanocomposites as novel photocatalyst for highly efficient degradation of organic contaminants. Compos. B Eng..

[7] Gatou M.-A., Syrrakou A., Lagopati N., Pavlatou E.A. (2024). Photocatalytic TiO2-based nanostructures as a promising material for diverse environmental applications: a review. Reactions.

[8] Oluwole A.O., Omotola E.O., Olatunji O.S. (2020). Pharmaceuticals and personal care products in water and wastewater: a review of treatment processes and use of photocatalyst immobilized on functionalized carbon in AOP degradation. BMC chemistry.

[9] Wang Y., Zhu J., Li M., Shao G., Wang H., Zhang R. (2023). Thermal properties of high-entropy RE-disilicates controlled by high throughput composition design and optimization. Mater. Des..

[10] Varghese R.J., Parani S., Thomas S., Oluwafemi O.S., Wu J. (2019).

[11] Ray S.S., Bousmina M. (2005). Biodegradable polymers and their layered silicate nanocomposites: in greening the 21st century materials world. Prog. Mater. Sci..

[12] Hori B.S., Rezvani M.A., Ardeshiri H.H., Panahiniya Z. (2023). Synthesis and characterization of new nanocomposite based on di-substituted Keggin-type phosphotungstate@ ceramic as a new and high-performance nanocatalyst for O2 evolution from water oxidation reaction. Int. J. Hydrogen Energy.

[13] Liu J., Xie S., Geng Z., Huang K., Fan L., Zhou W., Qiu L., Gao D., Ji L., Duan L. (2016). Carbon nitride supramolecular hybrid material enabled high-efficiency photocatalytic water treatments. Nano Lett..

[14] Nidheesh P., Khatri J., Singh T.A., Gandhimathi R., Ramesh S. (2018). Review of zero-valent aluminium based water and wastewater treatment methods. Chemosphere.

[15] D'Cruz B., Amin M.O., Al-Hetlani E. (2021). Polyoxometalate-based materials for the removal of contaminants from wastewater: a review. Ind. Eng. Chem. Res..

[16] Thostenson E.T., Li C., Chou T.-W. (2005). Nanocomposites in context. Compos. Sci. Technol..

[17] Gobi K., Mashitah M., Vadivelu V. (2011). Development and utilization of aerobic granules for the palm oil mill (POM) wastewater treatment. Chem. Eng. J..

[18] Khalafi N., Rezvani M.A., Jafarian V. (2023). Facile synthesis of new hybrid nanocomposite sandwich‐type polyoxometalate@ lead (II) oxide@ polyvinyl alcohol as an efficient and reusable amphiphilic nanocatalyst for ODS of of real fuel. Adv. Powder Technol..

[19] Khan R., Tariq M., Shaaban I.A., Assiri M.A., Bhatti M.H., Asif H.M. (2023). Synthesis of multi fluorine containing polyoxometalate sandwich type compound with drug delivery, DNA interaction and protein binding studies. Polyhedron.

[20] Zeb W., Altaf A., Aamir M., Baig N., Baig I., Nafady A., Sharif M., Sher M., Sohail M. (2022). Enhanced photoelectrochemical performance of P-doped g-C3N4/Zn0. 5Cd0. 5S heterojunction photocathode for water splitting. J. Saudi Chem. Soc..

[21] Vilà N., de Oliveira P., Walcarius A., Mbomekalle I.M. (2019). pH-modulated ion transport and amplified redox response of Keggin-type polyoxometalates through vertically-oriented mesoporous silica nanochannels. Electrochim. Acta.

[22] Tézé A., Cadot E., Bereau V., Herve G. (2001). About the Keggin isomers: crystal structure of [N (C4H9) 4] 4-γ-[SiW12O40], the γ-isomer of the Keggin ion. Synthesis and 183W NMR characterization of the mixed γ-[SiMo2W10O40] n-(n= 4 or 6). Inorg. Chem..

[23] Shojaei A.F., Rezvani A.M., Heravi M. (2011). A green, reusable and highly efficient solid acid catalyst for the oxidation of aldehydes to the corresponding carboxylic acids using H2O2 and KMnO4: H5PV2Mo10O40 (10-molybdo-2-vanadophosphoric heteropolyacid). J. Serb. Chem. Soc..

[24] Bijelic A., Rompel A. (2015). The use of polyoxometalates in protein crystallography–An attempt to widen a well-known bottleneck. Coord. Chem. Rev..

[25] Mahmoodi N.M., Rezvani M.A., Oveisi M., Valipour A., Asli M.A. (2016). Immobilized polyoxometalate onto the modified magnetic nanoparticle as a photocatalyst for dye degradation. Mater. Res. Bull..

[26] Shojaei A.F., Rezvani M.A., Heravi M. (2011). H_5_PV_2_Mo_10_O_40_ as an efficient catalyst for the oxidation of thiols to the corresponding disulfides using hydrogen peroxide as the oxidant. J. Serb. Chem. Soc..

[27] Behzadi M., Jarollahi S., Ahsani Irvani M., Ghanbari D. (2022). Green synthesis and antibacterial activity of silver nanoparticles using dracocephalum moldavica leaves extract. Journal of Nanostructures.

[28] Rezvani M.A., Khandan S. (2018). Synthesis and characterization of a new nanocomposite (FeW_11_V@CTAB-MMT) as an efficient heterogeneous catalyst for oxidative desulfurization of gasoline. Appl. Organomet. Chem..

[29] Rezvani M.A., Hadi M., Mirsadri S. (2020). Synthesis of new nanocomposite based on nanoceramic and mono substituted polyoxometalate, PMo_11_Cd@MnFe_2_O_4_, with superior catalytic activity for oxidative desulfurization of real fuel. Appl. Organomet. Chem..

[30] Wan S., Yu K., Wang L., Su Z., Zhou B. (2015). Assembly of sandwich-type 3-D supramolecular coordination polymers based on hexa-molybdenum chain and {PMo12O40} heteropolyanion. Inorg. Chem. Commun..

[31] Sundius T., Brandán S.A. (2023). Structural and vibrational characterization of di-hydrated hydrochloride tacrine combining DFT with SQMFF approach. Heliyon.

[32] Amini R., Nabiyouni G., Jarollahi S. (2021). Removal of azo dyes pollutants: photo catalyst and magnetic investigation of iron oxide-zinc sulfide nanocomposites. Journal of Nanostructures.

[33] Xu P., Liu X., Zhao Y., Lan D., Shin I. (2023). Study of graphdiyne biomimetic nanomaterials as fluorescent sensors of ciprofloxacin hydrochloride in water environment. Desalin Water Treat.

[34] Fang T., Chen X., Wang M., Wang Y., Liao L., Li B. (2020). Cuprous oxide/titanium dioxide composite photocatalytic decolorization of reactive brilliant red X-3B dyes wastewater under visible light. Res. Chem. Intermed..

[35] Almeida Guerra W.N., Teixeira Santos J.M., Raddi de Araujo L.R. (2012). Decolorization and mineralization of reactive dyes by a photocatalytic process using ZnO and UV radiation. Water Sci. Technol..

[36] Zhu C., Li Y., Yang Y., Chen Y., Yang Z., Wang P., Feng W. (2020). Influence of operational parameters on photocatalytic decolorization of a cationic azo dye under visible-light in aqueous Ag3PO4. Inorg. Chem. Commun..

